# Supplementary figures and images for: The Transcriptional landscape of Streptococcus pneumoniae TIGR4 reveals a complex operon architecture and abundant riboregulation critical for growth and virulence
Source: PLoS Pathog. 2018 Dec 5;14(12):e1007461. doi: 10.1371/journal.ppat.1007461 (PMC6296669; doi:10.1371/journal.ppat.1007461)

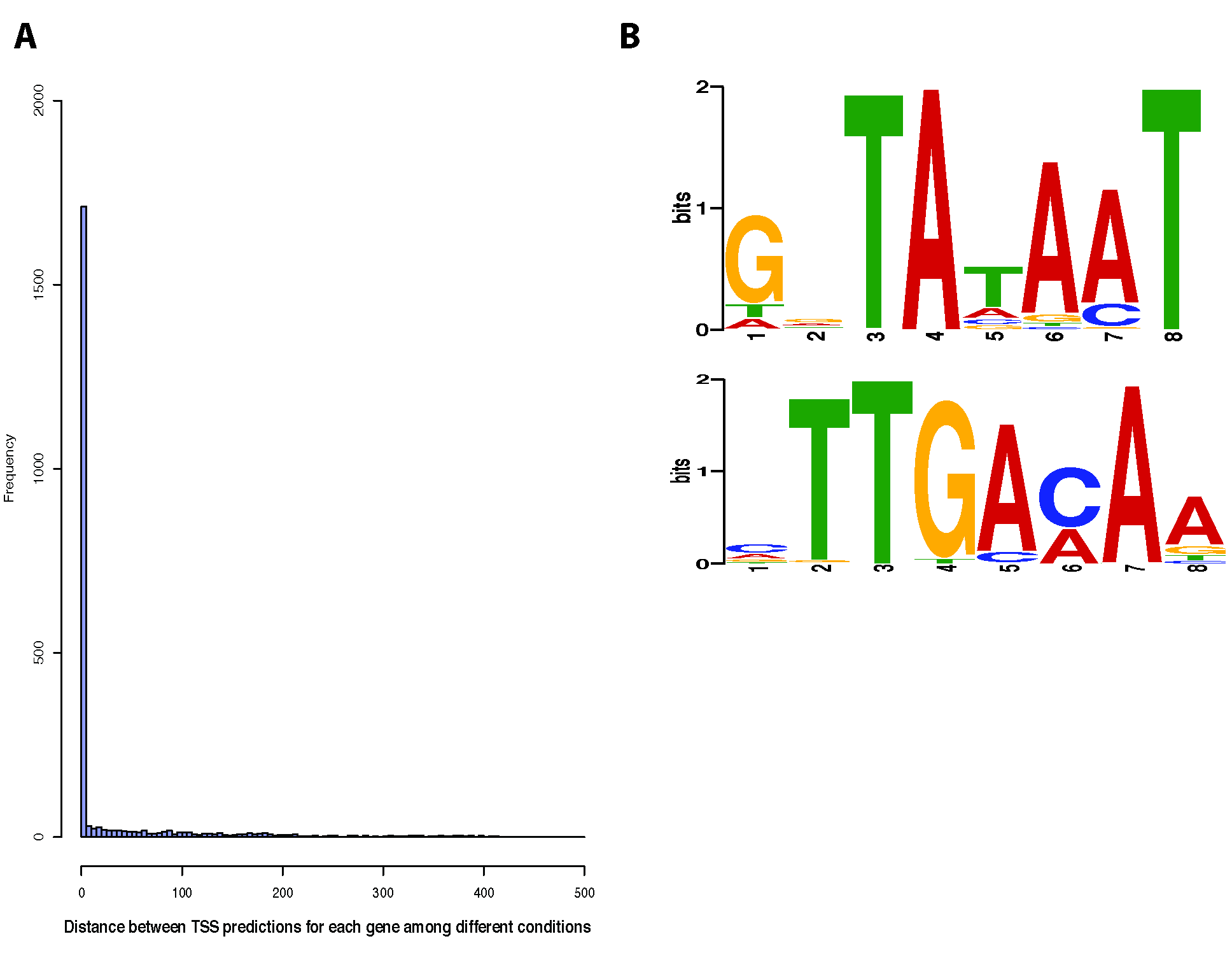

Supplement: S1 Fig — A. Frequency distribution of the distance between the TSS position predicted for each gene between conditions. This also takes into account whether TSSs were predicted. Majority of the predictions match exactly or are very close to one another between the conditions providing confidence in the TSS predictions from the pooled data. B. Predicted TSSs show enrichment of the TATA box at the -10 and the TTGACA at the -35 positions respectively as identified by MEME [72]. (TIF) [file ppat.1007461.s001.tif]

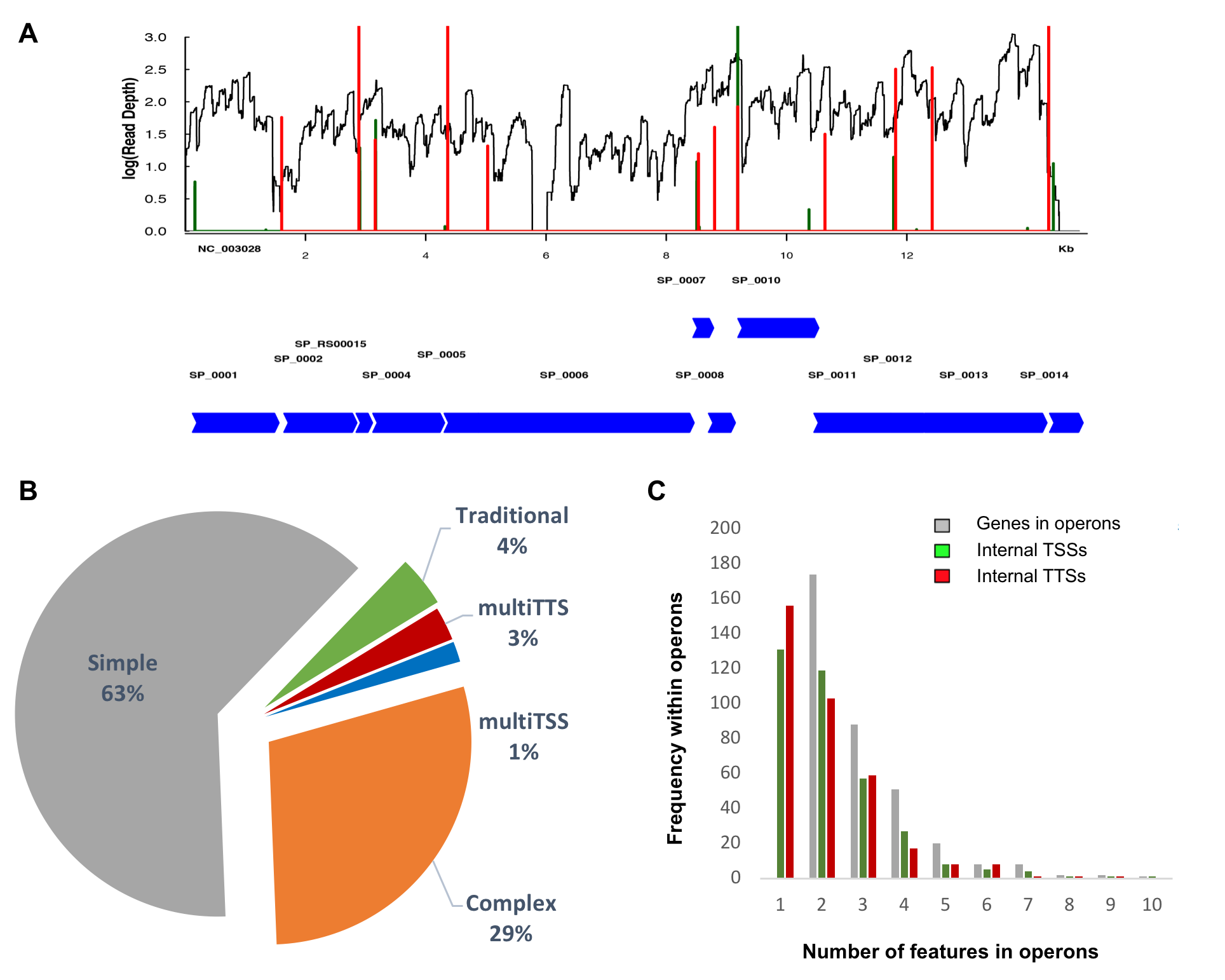

Supplement: S2 Fig — (A) StringTie reconstructed transcript grouping genes SP_0001 –SP_0014. Log transformed paired-end RNA-Seq coverage map across this region. Red and Green lines represent the predicted TSSs and TTSs (B) The pie chart describes the distribution of the types of operons present in T4 identified using single-end RNA-Seq analyzed with Rockhopper. A total of 474 multigene and 773 single gene operons were identified, which can be divided up in 63% simple operons (single gene transcriptional units with a single TSS and TTS; gray), 29% complex operons (multi-gene operons with multiple TSSs and TTSs; orange), 4% traditional operons (multi-gene operon with a single TSS and TTS; green), 1% multiTSS operons (blue), and 3% multiTTS operons (red). (C) The clustered histogram describes the distribution of genes and transcriptional features in non-traditional operons described by Rockhopper, where gray represents the numbers of genes in the multigene operons, green represents the number of TSSs within operons, red represents the number of TTSs within operons. Two-gene operons are found most frequently in the non-traditional operons with one internal TSS and TTS. (TIFF) [file ppat.1007461.s002.tiff]

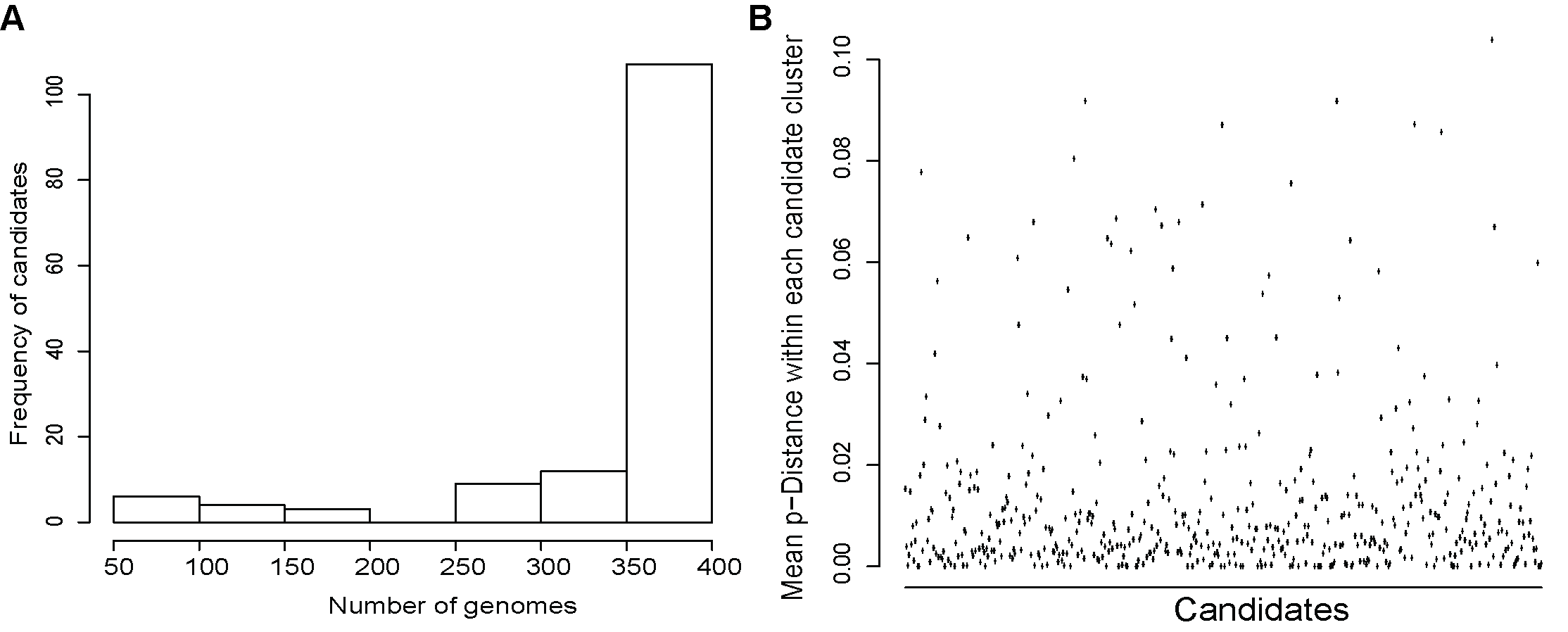

Supplement: S3 Fig — A. Frequency distribution of the candidates across the surveyed genomes. B. Conservation of the candidates as a measure of the mean p-Distance within each candidate cluster. (TIF) [file ppat.1007461.s003.tif]

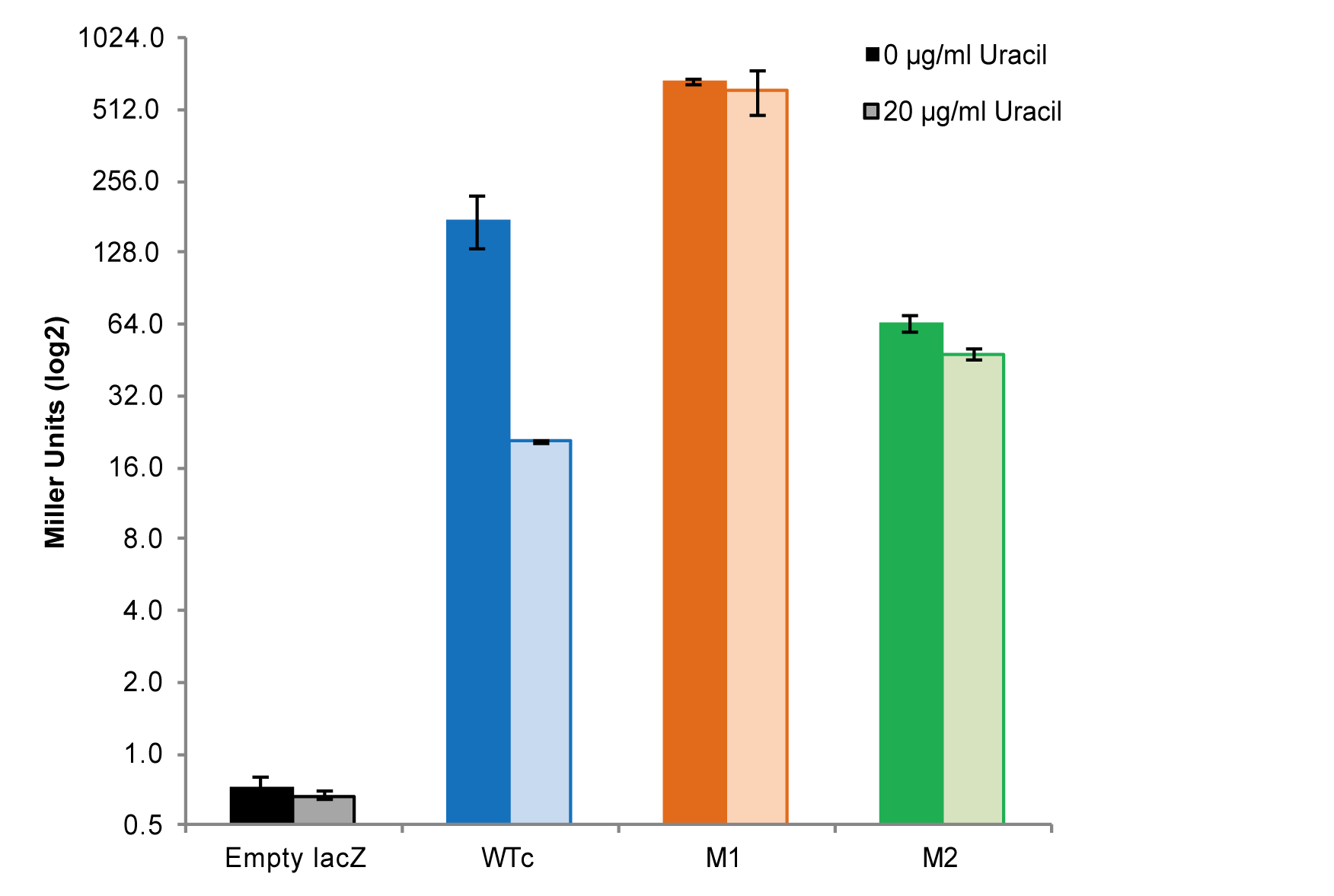

Supplement: S4 Fig — The activity of the wild type pyrR decreases in the presence of uracil, while M1 is insensitive to the ligand. Activity of mutant M2 results in increased Miller units even in the presence of uracil. Empty lacZ represents a β-galactosidase reporter construct without a regulatory region upstream, which was used as a negative control. Error bars represent standard error of the mean across three technical replicates. (TIF) [file ppat.1007461.s004.tif]
