# Supplementary material for: The Transcriptional landscape of Streptococcus pneumoniae TIGR4 reveals a complex operon architecture and abundant riboregulation critical for growth and virulence
Source: PLoS Pathog. 2018 Dec 5;14(12):e1007461. doi: 10.1371/journal.ppat.1007461 (PMC6296669; doi:10.1371/journal.ppat.1007461)
Supplement: S5 Table — List of adapters used for 5’ end-Seq. Highlighted in red are the barcode sequence (5’-3’). (DOCX) [file ppat.1007461.s009.docx]

**S5 Table: RNA adapters with barcode for 5’ end-seq**

| **Adapter** | **Sequence** |
| --- | --- |
| RNABC01_RC | 5’-SpC3-CUACACGACGCUCUUCCGAUCUAAGCAAU-3’ |
| RNABC02_RC | 5’-SpC3-CUACACGACGCUCUUCCGAUCUAAUUCAU-3’ |
| RNABC03_RC | 5’-SpC3-CUACACGACGCUCUUCCGAUCUACAAGUU-3’ |
| RNABC04_RC | 5’-SpC3-CUACACGACGCUCUUCCGAUCUACAGCCU-3’ |
| RNABC05_RC | 5’-SpC3-CUACACGACGCUCUUCCGAUCUACCUGAU-3’ |
| RNABC06_RC | 5’-SpC3-CUACACGACGCUCUUCCGAUCUACUAAUU-3’ |
| RNABC07_RC | 5’-SpC3-CUACACGACGCUCUUCCGAUCUACUCAAU-3’ |
| RNABC08_RC | 5’-SpC3-CUACACGACGCUCUUCCGAUCUAGACCAU-3’ |
| RNABC09_RC | 5’-SpC3-CUACACGACGCUCUUCCGAUCUAUAAACU-3’ |
| RNABC10_RC | 5’-SpC3-CUACACGACGCUCUUCCGAUCUAUACAUU-3’ |
| RNABC11_RC | 5’-SpC3-CUACACGACGCUCUUCCGAUCUAUCAAUU-3’ |
| RNABC12_RC | 5’-SpC3-CUACACGACGCUCUUCCGAUCUAUCCACU-3’ |
| RNABC19_RC | 5’-SpC3-CUACACGACGCUCUUCCGAUCUCACGAAU-3’ |
| RNABC23_RC | 5’-SpC3-CUACACGACGCUCUUCCGAUCUCCCGAUU-3’ |
| RNABC24_RC | 5’-SpC3-CUACACGACGCUCUUCCGAUCUCCGACUU-3’ |
| RNABC34_RC | 5’-SpC3-CUACACGACGCUCUUCCGAUCUGGCCUCU-3’ |
| RNABC36_RC | 5’-SpC3-CUACACGACGCUCUUCCGAUCUGUACCCU-3’ |
| RNABC37_RC | 5’-SpC3-CUACACGACGCUCUUCCGAUCUGUUUGCU-3’ |
| RNABC51_RC | 5’-SpC3-CUACACGACGCUCUUCCGAUCUUGGUCCU-3’ |
| RNABC52_RC | 5’-SpC3-CUACACGACGCUCUUCCGAUCUUGUUACU-3’ |
| RNABC53_RC | 5’-SpC3-CUACACGACGCUCUUCCGAUCUUUCCCGU-3’ |
