# Supplementary material for: The Transcriptional landscape of Streptococcus pneumoniae TIGR4 reveals a complex operon architecture and abundant riboregulation critical for growth and virulence
Source: PLoS Pathog. 2018 Dec 5;14(12):e1007461. doi: 10.1371/journal.ppat.1007461 (PMC6296669; doi:10.1371/journal.ppat.1007461)
Supplement: S6 Table — (DOCX) [file ppat.1007461.s010.docx]

**S6 Table: Primers used in this study**

| **Primer Name** | **Sequence (5’ to 3’)** | **Description** |
| --- | --- | --- |
| 3Tr3_RC+RH | CAGACGTGTGCTCTTCCGATCTNNNNNN | Primer used 5’ end-Seq library preparation |
| SP2204_qPCR_F | TCTGCAATCATCTCAGCGTGAG | Primer for qRT-PCR targeting SP_2204 |
| SP2204_qPCR_R | TCTAGCCAAAGAAGCGACTGC |  |
| SP0178_qPCR_F | AGGTGTTTTCCCGAAGTGAC | Primer for qRT-PCR targeting SP_0178 |
| SP0178_qPCR_R | GCCCACATGCTGAGAGAAATG |  |
| SP0716_qPCR_F | TCAGCCAGGTTTGACTGTTG | Primer for qRT-PCR targeting SP_0716 |
| SP0716_qPCR_R | TTGTCCCCGCAAAAAGTTCC |  |
| SP1782_qPCR_F | AAATGTGGACCGCTTTGGAG | Primer for qRT-PCR targeting SP_1782 |
| SP1782_qPCR_R | TGTCACATCCACCGTGTCAG |  |
| SP1278_qPCR_F | ATAACTTCGCGGTCTGTCAC | Primer for qRT-PCR targeting SP_1278 |
| SP1278_qPCR_R | CCACCGAATCCAAGAACGTTTG |  |
| SP_1278_F1 | TGATGGAAGTCGTAATCGT | Primers to amplify the left homology arm |
| SP_1278_R1 | CATCAAGCTTATCGATACCGCTTGATTTTAAGCATGCTTT |  |
| SP_1278_F2 | GAAGAAGGTTTTTATATTACAGCTCCACAAAAAGTTTCAATGTAGCA | Primers to amplify the right homology arm |
| SP_1278_R2 | TATGGAGCCAGTTGAGTTA |  |
| SP_1278_M1_F | CTTGCCTGCCTCACGACAGGTTTAAAG | Mutagenesis primers for M1 pyrR RNA mutant |
| SP_1278_M1_R | CTTTAAACCTGTCGTGAGGCAGGCAAG |  |
| SP_1278_M2_F | CCTCCAAAAAGAATGGTCTCCTTAAACAA | Mutagenesis primers for M2 pyrR RNA mutant |
| SP_1278_M2_R | TTGTTTAAGGAGACCATTCTTTTTGGAGG |  |
| SP_1278_M3_F | CCTTAAACAAGGAGACTTGACAGGTTTAAAGGAATATTTAG | Mutagenesis primers for M3 pyrR RNA mutant |
| SP_1278_M3_R | CTAAATATTCCTTTAAACCTGTCAAGTCTCCTTGTTTAAGG |  |
| SP_1278_F0 | CCAGCGAAAAAGAGTTCA | Sequencing primers |
| SP_1278_R0 | ATTGTTCTTGCTTCGAGTC |  |
