# Supplementary material for: The Transcriptional landscape of Streptococcus pneumoniae TIGR4 reveals a complex operon architecture and abundant riboregulation critical for growth and virulence
Source: PLoS Pathog. 2018 Dec 5;14(12):e1007461. doi: 10.1371/journal.ppat.1007461 (PMC6296669; doi:10.1371/journal.ppat.1007461)
Supplement: S1 Methods — (DOCX) [file ppat.1007461.s011.docx]

**Supplemental Methods:**

*5’ end-seq library preparation*

1 ug total RNA was DNase-treated and cleaned with 2X paramagnetic SPRI beads and eluted with 13 μl of nuclease-free H_2_O. Cleaned RNA was incubated with 20 U of 5’ polyphosphatase (or Nuclease-free H_2_O for mock reaction) for 1 h at 37^o^C to generate 5’ monophosphatse RNAs and further cleaned using 2X paramagnetic SPRI beads. RNA adapters carrying barcodes (for specific conditions) were ligated to the 5’ end of RNA by mixing 5 μl of RNA solution with 1 μl of 100 μM adapter solution (sequence in S6 Table), 2 μl of 10X T4 ligase buffer, 1.8 μl of DMSO, 0.2 μl of 100mM ATP, 8 μl of 50% PEG 8000, 0.3 μl of RNase inhibitor, murine (40U/ul) and 1.8 μl of T4 RNA ligase 1 enzyme. The reaction was incubated for 1.5 h at 22^o^C and cleaned to with 2.2X paramagnetic SPRI beads. The RNA was fragmented in fragmentation buffer (Ambion) by incubating at 72^o^C for 1.5 min. Barcoded RNA samples from different conditions were cleaned using Zymo column and pooled. Ribosomal RNA was depleted using Ribo-Zero rRNA removal kit (Illumina) according to manufacturer’s instructions. First strand cDNA was synthesized by using AffinityScript multiple temperature cDNA synthesis kit (Agilent), as per manufacturer's instructions. The primer used was a illumina 3’ adapter-linked random hexamer primer, 3Tr3_RC+RH (sequence in S6 Table). To degrade the RNA template, 10% reaction volume of freshly made 1N NaOH was added, incubated at 70^o^C for 12 min and neutralized with 4 μl of freshly made 0.5M acetic acid. The RT reaction was further cleaned using 2X SPRI beads to remove primer. The cleaned library was amplified using Q5 high-fidelity DNA polymerase with Illumina index primers for 17 amplification cycles. The PCR reaction was first cleaned with 1.5X SPRI beads to remove reaction buffers and primers. cDNA libraries sized 150-500 bp was selected by performing double selection with SPRI beads. First selection (0.5X SPRI beads) removed larger fragments (more than 500-600 bp) while the next selection with 1X SPRI beads removed smaller fragments (more than 100bp).

*β-Galactosidase reporter strain construction and activity assay*

To generate pyrR RNA *lacZ* reporter strains, the native β-galactosidase (*bgaA*) locus of *S. pneumoniae* TIGR4 was replaced with a pyrR regulatory element (or their mutant) construct that was translationally fused to *lacZ* amplified from a *B. subtilis* integration vector pDG1728 [1] expressing a soluble β-galactosidase protein. *S. pneumoniae* pyrR mutant *lacZ* reporter strains were cultured in CDM (+/- 20 μg/ml Uracil) to mid-log. Cells were resuspended in Z buffer (50 mM Na2HPO4, 40 mM NaH2PO4, 10 mM KCl, 1 mM MgSO4, 50 mM 2-mercaptoethanol) and β-galactosidase activity assay was performed as previously described [2].

References-

1. Guérout-Fleury A, Frandsen N, Stragier P. Plasmids for ectopic integration in *Bacillus subtilis*. Gene. 1996;180: 57–61.

2. Miller J. A short course in bacterial genetics. Cold Spring Harbor Laboratory Press; 1992.
